# The Effect of 12-Weeks Recreational Football (Soccer) for Health Intervention on Functional Movement in Older Adults

**DOI:** 10.3390/ijerph192013625

**Published:** 2022-10-20

**Authors:** Michael J. Duncan, Sophie Mowle, Mark Noon, Emma Eyre, Neil D. Clarke, Mathew Hill, Jason Tallis, Mikko Julin

**Affiliations:** 1 Centre for Sport, Exercise, and Life Sciences, Coventry University, Coventry CV1 5FB, UK; 2 Research, Development and Innovation (RDI) Unit, Laurea University of Applied Sciences, 02650 Espoo, Finland

**Keywords:** walking football, older adults, physical activity, motor skill, functional fitness

## Abstract

There is growing evidence that recreational football offers health benefits for older adults and an important pathway for physical activity for older adult groups. Despite anecdotal evidence that recreational football is beneficial for older adults, no empirical data are available to support this assertion. This study addressed this issue and examined the effects of a 12-week recreational football intervention on the functional fitness of older adults. Using a pre–post case–control design, thirteen males, aged 61–73 years (mean age ± SD = 66 ± 4 years) undertook a twice-weekly, 12-week recreational football for health intervention, and were matched with a control group, comprising thirteen males, aged 62–78 years (mean age ± SD = 66 ± 4 years) who maintained their typical exercise habits during the intervention period. Pre- and postintervention, participants underwent assessment of functional fitness, using the Rikli and Jones functional fitness battery as well as an assessment of body fatness, via bioelectrical impedance analysis and dominant handgrip strength using handgrip dynamometry. Results from a series of 2 (pre–post) X 2 (intervention vs. control) repeated-measures ANOVAs indicate significant pre–post X group interactions for the 30-second chair stand (*p* = 0.038, Pƞ^2^ = 0.168), 8-foot timed up and go (*p* = 0.001, Pƞ^2^ = 0.577) and 6 min walk test (*p* = 0.036, Pƞ^2^ = 0.171). In all cases, performance improved significantly after the intervention for the football intervention group but not the control group. There were no significant differences in the 30 s arm curl test or dominant handgrip strength (*p* > 0.05). There was a non-significant trend (*p* = 0.07, Pƞ^2^ = 0.127) towards a pre–post X group interaction for body fatness, showing a decreased percent body fat for the intervention group over the control group. The results of the present study demonstrate the utility of recreational football as a physical activity intervention in older adults to improve functional movement.

## 1. Introduction

The concept of football as medicine is now well-established in the literature [1]. Football is a broad-spectrum exercise that improves cardiovascular and musculo-skeletal fitness, and reduces the risk of cardiovascular disease, falls and fractures [2]. Importantly, at grassroots levels, football represents an attractive opportunity to engage populations in physical activity on a habitual basis, due to its relatively low cost, availability of related facilities and, often, older adults’ prior experiences in football in their younger years. Small-sided walking football in particular has been shown, in some studies, to improve health-related indices in older adults [3,4]. As a consequence, there has been a recent influx in the use of football for health benefits [5]. Walking football in older adult groups (ages 60 years and older) has gained popularity due to the lower intensity and perception that it is safer than regular football [6,7,8]. Although popular as a recreational physical activity in the older adult population, there are relatively few studies documenting the effect of walking football on health parameters in older adult groups. In one study, Arnold et al. [6] reported mixed findings across fitness and physiological parameters. Walking football significantly increased time to exhaustion in an incremental exercise test following a 12-week intervention in a sample of 10 males aged 50 years and older, but with no change in peak blood lactate and a slight reduction in peak heart rate, suggesting enhanced exercise tolerance [6]. Thus, while the programme enhanced exercise tolerance, it is possible that the intensity of the exercise programme was insufficient to significantly enhance aerobic fitness as no significant change was reported in VO_2_ peak. However, the 12-week walking football programme, in the form of one 2 h training session per week, positively altered a range of anthropometrical parameters, with large 11% and 9% reductions in body fat mass and percentage body fat, respectively, being observed along with moderate reductions in body mass index (BMI; 3%). Furthermore, Hubball and Reddy [7] reported that competitive walking football was enjoyable, could be played more frequently with less ‘strain’ than regular football, and the emphasis on passing had team-building benefits for Canadian high-performance veteran football players. However, despite these sociological benefits, Reddy et al. [8], based on the same protocol as Hubbal and Reddy [7], observed that walking football was found to be engaging, sustainable for older adults and moderately intensive, but without health and cognitive benefits, possibly due to the low exercise intensity as running is not permitted.

Unlike walking football, which is restricted in terms of the type and lower intensity of locomotion, recreational football is typically more dynamic and of higher intensity [1]. Running is permitted, and intensity of exercise is self-regulated, making it acceptable and tolerable for older adults. There is a growing body of evidence highlighting the beneficial effects of participation in recreational football on physical fitness and health status. Many individual studies have reported recreational football to be an effective type of physical activity with positive effects on hypertension in middle-aged men [9] and women [10], on cardiovascular and metabolic responses in patients with type 2 diabetes [11], on heart function [12], on physical capacity [13], on muscle mass in patients with prostate cancer [12] and on bone mineral density and mass [14]. Despite this, and despite anecdotal assertions that recreational football is safe for older adults to undertake, no empirical data are available that examine if participation in recreational football enhances functionality in older adults. As functional performance is a key factor in older adults’ health and engagement in physical activity [15,16,17], understanding if this form of football improves functional movement is a needed first step before it could be suggested as an effective physical activity intevention for older adult health. The present study sought to address this issue by examining the effects of a 12-week recreational football health intervention on functional movement and body fatness in older adults. 

## 2. Materials and Methods

This study used a pre–post, case–control design whereby two matched groups (football intervention vs. control) were recruited to take part in the study. Groups were matched for sex and age. The procedure for conduct of the case–control study, including allocation of case–controls, followed that recommended by the UK Health Security Agency [18] for such studies. All assessments took place in a community sports facility comprising a sports hall and outdoor artificial football pitch. 

### 2.1. Participants

**Figure 1:**
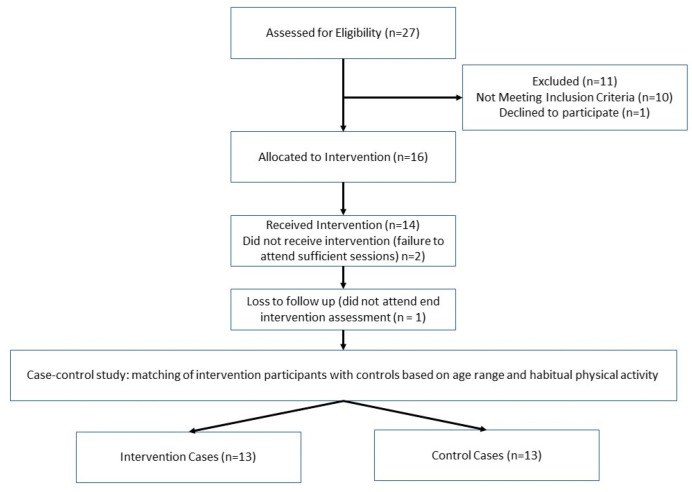
CONSORT flow diagram for recruitment using case–control studies.

Following institutional ethics approval (approval number P92159) and written informed consent, thirteen males aged 60–80 years (mean age ± SD = 66 ± 4 years, min–max = 61–73 years) undertook a 12-week recreational football for health intervention. The intervention group was matched with a control group (*n* = 13 males, aged 60–80 years (mean age ± SD = 66 ± 4 years, min–max = 62–78 years) who did not alter their exercise habits during the 12-week intervention period. Participants were recruited from the local community in Coventry and Warwickshire. To be eligible, participants had to be considered ‘apparently healthy’ with no evidence of cardiovascular incident within the past 12 months, no existing or significant past medical history of vascular disease, cancer, diabetes, kidney, pulmonary, thyroidal disease, osteoporosis or history of falls, no known cognitive impairment, no neuromuscular disorder or injury, no use of corticosteroids during the last six months and no presence of uncontrolled hypertension. The exclusion criteria were set within the regulations of the institutional ethics committee approving the project regarding undertaking exercise interventions with older adults. A CONSORT flow diagram for recruitment using case–control studies is presented in Figure 1.

### 2.2. Procedure

Intervention and control groups both undertook the same assessment before and after the 12-week intervention period. Participants undertook assessments of functional fitness, using the Rikli and Jones Senior Fitness test [19], height and body mass, from which body mass index (BMI, kg/m^2^) was determined, and percentage body fatness [20] using bioelectrical impedance analysis (Tanita BF-350, Tanita Inc, Tokyo, Japan). Participants adhered to recommended guidelines for accurate assessment of body fatness using bioelectrical impedance analysis including no drinking for 4 h prior to the test, no exercise for 12 h prior to the test, urination 30 min prior to the test and no alcohol consumption for 48 h before the test [21]. Habitual physical activity was determined using the International Physical Activity Questionnaire (Long Form). The IPAQ has been extensively used, and administration of this form was conducted according to recommended protocols [22]. Data are presented as (MET/Min week^−^^1^) in line with recommendations for scoring of the IPAQ measure (IPAQ) as this provides a measure of total weekly physical activity [23]. Before the intervention, there was no significant difference in habitual physical activity between participants in the intervention and participants in the control group (*p* = 0.846). Participants in the control group were also asked to confirm, at the post-test, they had not changed their habitual activities during the course of the intervention.

#### 2.2.1. Assessment of Functional Movement

Functional movement was assessed using tests from the Rikli and Jones [19,24] Senior Fitness Test, comprising: six-minute walk test (6MWT), 8-foot timed get up and go (TUG), arm curl (AC) and 30 s chair stand (CS). All procedures followed those described previously for administration of the Senior Fitness Test [19]. The TUG comprised the number of seconds required to get up from a seated position, walk 8 feet as fast as possible, turn and return to the seated position. For the AC, the number of bicep curls that were completed in 30 s holding a hand weight of 2.27 kg for females and 3.63 kg for males was recorded. The CS test comprised the number of full stands that could be completed, from a seated position, in 30 s with arms folded across the chest. For the 6MWT, participants were instructed to walk as quickly as possible for 6 min up and down a 20 m walkway marked off in 2 m segments and were informed that they could slow down or rest if necessary. Standardised encouragement was given each minute during the tests. The distance walked was recorded and used for analysis. Isometric handgrip strength on the dominant hand was also assessed using a handgrip dynamometer (Takei instruments, Tokyo, Japan) and taken as a measure of muscle strength. With the exception of the 6MWT, where participants undertook one trial at the end of the assessment session, participants undertook three trials of each assessment with the best score in each being used for analysis.

#### 2.2.2. Recreational Football for Health Intervention

All participants in the intervention group undertook a 12-week, twice-weekly recreational football for health programme based on those previously used [25] and following recommended guidelines for administration of recreational football for heath programmes in older adult groups [26]. Each session comprised a 15 min warm up using a RAMP (Raise, Activate and Mobilise, Potentiate, [27]) protocol, followed by a series of six four-minute small-sided games comprising 4 × 4, or 3 × 3 participant numbers with a four-minute rest period between games and a five-minute cool down for a total of 60 min per session. The rules of the small-sided games were modified to include no placing the foot on top of the ball and no physical contact between players (tackling and pushing) as per guidelines for this form of physical activity [27]. There were no throw ins within the small-sided games, with restarts taking place via a pass into the playing area. The intervention took place on an artificial macadam surface measuring approximately 30 × 15 metres. During each session, at the end of each small-sided game, individual exercise intensity was assessed using the Borg 6–20 rating of perceived exertion (RPE [27]) scale and following recommended guidelines for administration and collation of exercise intensity data [28].

#### 2.2.3. Data Analysis

In order to examine any differences in functional fitness performance and anthropometric parameters between intervention and control groups before and after the intervention, a series of 2 (pre–post) X 2 (Intervention vs. control) repeated-measures analysis of variance (ANOVA) were used. Assumptions of normality of data and homogeneity of variance were met to enable use of parametric statistics. Furthermore, 95% confidence intervals (95% CI) were calculated, and partial η^2^ was used as a measure of effect size with values of <0.10, 0.10–0.24, 0.25–0.39 and ≥0.40 defined as trivial (<0.10), small, moderate or large, respectively. IBM SPSS Statistics (SPSS, v25, IBM Corp, Armonk, NY, USA) was used for all analysis. The alpha value was a priori set at *p* < 0.05 for all tests.

## 3. Results

**Figure 2:**
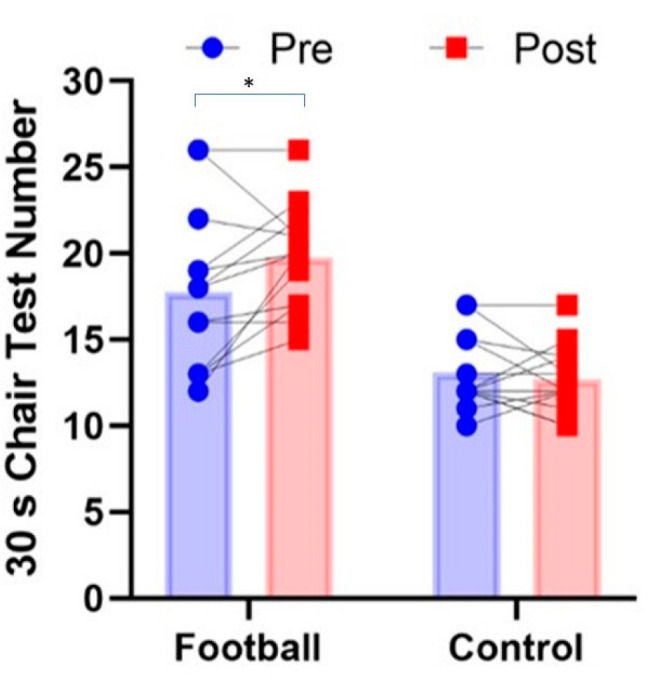
Group mean and individual performance for 30 s chair stand performance (number of repetitions) before and after in recreational football for health intervention group and matched (* *p* < 0.05).

**Figure 3:**
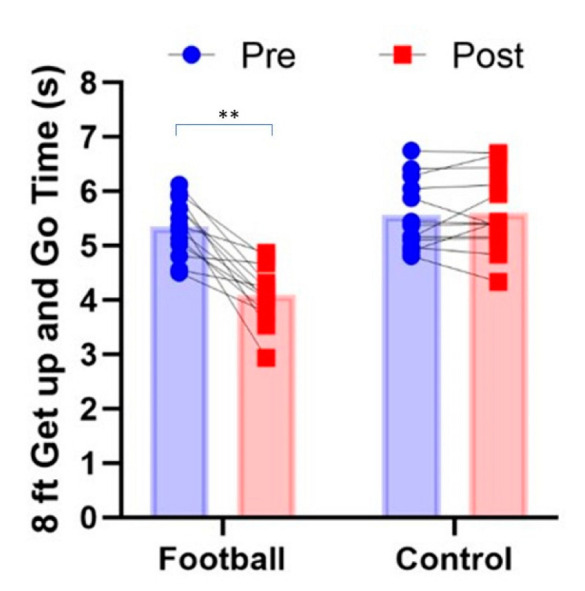
Group mean and individual time for 8-foot get up and go performance (s) before and after in recreational football for health intervention group and matched controls (** *p* < 0.01).

**Figure 4:**
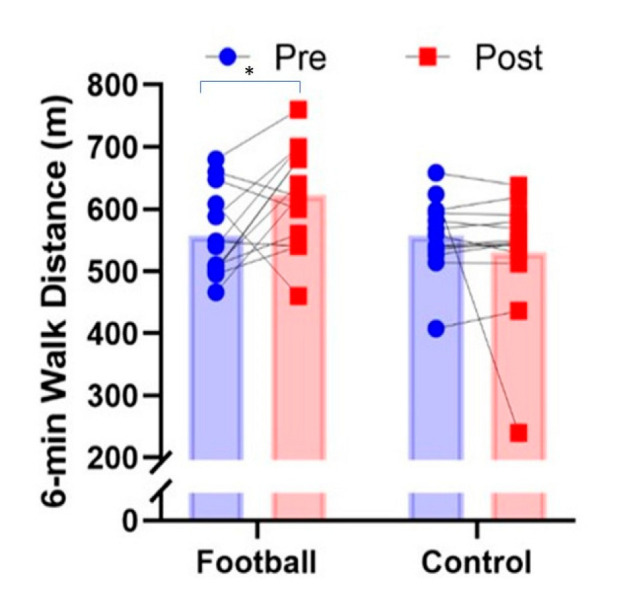
Group mean and individual performance for 6 min walk test performance (m) before and after in recreational football for health intervention group and matched controls (* *p* < 0.05).

**Figure 5:**
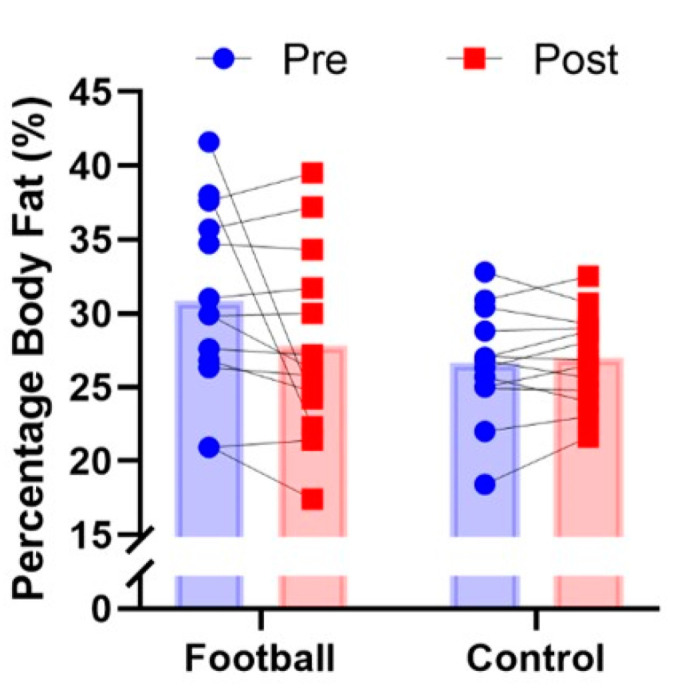
Group mean and individual performance for percentage body fat (%) before and after in recreational football for health intervention group and matched controls.

Mean, SD and 95% CIs for all variables before and after for intervention and control groups are presented in Table 1. Significant pre–post X group interactions were observed for the 30 s chair stand test (*p* = 0.038, Pƞ^2^ = 0.168, Figure 2), 8-foot get up and go test (*p* = 0.001, Pƞ^2^ = 0.577, Figure 3) and 6 min walk test (*p* = 0.036, Pƞ^2^ = 0.171, Figure 4). In all cases, performance improved significantly after the intervention for the football intervention group but not the control group. No significant differences in the 30 s arm curl test or dominant handgrip strength (*p* > 0.05) were observed. A non-significant trend (*p* = 0.07, Pƞ^2^ = 0.127, Figure 5) towards a pre–post X group interaction for body fatness, showing decreased percent body fat for the intervention group over the control group. In regard to perceived exercise intensity assessed at the end of each of the small-sided games within each intervention session, the mean SD for RPE was 11.7 ± 0.8 (95CIs: 10.4–12.6), reflecting intensity that was moderate in nature or ‘somewhat hard’ in the view of the participants. Among participants, RPE scores fell between 10 and 15 on the 6–20 Borg scale, suggesting intensity ranged from ‘light’ to ‘hard’ for different participants.

## 4. Discussion

The current study demonstrates the effect of recreational football for health as a physical activity intervention to enhance functional fitness in older adults. The results of this study suggest that such an intervention results in significant improvement in functional movement performance that is reflective of lower body muscular endurance, agility and aerobic endurance performance via increased improvements in the 30 s chair stand, 8-foot get up and go and 6 min walk test performances before and after in the football intervention group, compared to a matched control group.

Such results would align with prior work, albeit in different populations, which suggests recreational football for health interventions enhance health-related indices [9,11,12,13,14]. The results of the present study also support the suggestions that small-sided football can improve health-related variables in older adults [3,4]. However, unlike prior empirical work in older adults [4], which has relied on pre–post designs with no control group, the current study used a matched case–control design where participants in the intervention group were matched with participants in a control group who did not undertake the intervention. We matched participants based on habitual physical activity levels but did not track habitual activity for either group during the intervention period. Likewise, up to this point, no study had documented the effect of recreational football interventions on functional movement in older adults. This latter point is important as age-related declines in muscle strength result in a reduction in functional performance [15]. This is particularly the case for functional movement tasks, which rely on muscle strength in the lower extremities (e.g., stair climbing) [29] as well as other motor tasks such as balance performance and walking [29].

Our results demonstrate that recreational football enhances functional movement in older adults. In older adult groups, habitual physical activity tends to be characterised by recreational activities such as walking and golf, which have a low-force demand [16]. Such activity may not provide sufficient overload to develop muscle strength, and thus functional benefit arising from typical recreational activity in community-dwelling older adult groups may be blunted [15]. We would argue there may be better methods to enhance functional performance and muscle strength in older adults than those suggested as typical by Chodzko-Zajko et al. [16]. This might include age-appropriate, group-based recreational sport, as demonstrated by the results of the present study and suggested by prior authors [1,3]. Likewise, research reporting the results from walking football intervention studies have suggested, similarly that it may not be sufficiently force demanding to result in significant health benefit [8]. Given that recreational football allows running and running generates significantly greater force than walking in older adults [29,30,31,32], it seems logical to suggest that the physical demands of recreational football for older adults are sufficient to improve performance in functional tasks.

There is one question regarding the core concept underpinning recreational football for health and the mechanisms by which it may be efficacious. Readers are referred to the key text by Krustrup and Parnell [1], which provides an in-depth overview of the development of the concept, and scientific basis, of football as medicine, including recreational football for health. However, in short, the core concept underpinning recreational football for health is the participation in a group sports activity that is multidirectional in nature and challenges multiple physical capacities, including the muscular and aerobic systems, challenges stability and decision making and occurs in a social environment [1]. Recreational football in particular, compared to, say, walking football, is based on the tenant that there is no reason to constrain movement opportunity and reduce exercise ‘load’ (i.e., running) by imposing a condition that participants must only walk if they are physically able to do more than walk [33]. Such an approach also increases motivation to engage in the activity and, alongside the interaction between participants, is purported to better increase adherence to the exercise intervention compared to other types of interventions, such as those solely involving resistance or aerobic exercise [3].

Prior research has also suggested that playing team sports might increase intrinsic motivation and enjoyment of physical activity [34], resulting in greater adherence to exercise intervention and thus larger magnitudes of change in the outcomes from undertaking exercise. Although we did not assess intrinsic motivation or enjoyment of the participants, it is possible the format of playing football in a team setting created the motivational climate for continued engagement in this activity in the present study. It is important, however, to highlight that the results presented in the current study are reflective of an already relatively active older adult population. For example, baseline values for the 6 min walk test for both the intervention and control population place them in the 50th percentile for this aspect of functional fitness [24]. Similarly, at baseline, all participants were classified, based on the IPAQ categories [25], as either moderately or highly physically active.

It is perhaps notable that participation in recreational football can improve functional fitness in an already active group, but it is also important to consider that the effect of recreational football may be more pronounced in older adults who are not physically active or fit at the onset of such activity. Conversely, given the participants in the present study were volunteers, future researchers should be mindful that recreational football as a concept may be more attractive for older adults who are already physically active, and if researchers wish to recruit inactive participants, a tailored recruitment strategy might be needed. The results of the present study would also align with conclusions drawn by Arnold et al.’s [6] work examining effects of recreational football on fitness performance in older adults, using a pre–post design, without a control group. Although not directly comparable with the present study as different markers of fitness were employed, and no control group was used in the study by Arnold et al. [6], both studies demonstrate positive impacts of recreational football on older adults’ participants. The present study, due to its use of a case–control design, comparing recreational football to a ‘usual practice’ control group, extends the understanding of the effect of recreational football on older adults’ functional performance in a way prior work has not.

As an adjunct, and in relation to the discussion of the physical ‘loading’ that older adults experience during recreational football, a question may be asked regarding potential for injury in this form of activity. Potential participants may also be apprehensive about undertaking a recreational football programme due to fear of injury. A range of factors, e.g., training history, body mass and fitness, may contribute to injury risk in older adults who undertake organised physical activity. While the current study does not address this issue of injury risk in recreational football, as part of our participant monitoring process, we recorded injuries that were sustained following every session. Recording and coding of injury data followed recommended protocols [35]. During the intervention, there were 11 injuries recorded in a total of 10 participants. The majority of injuries were graded as mild-to-moderate, such as minor strains of the lower body (calf, thigh, groin) with an overall injury incidence per hour of 0.32, which is regarded as low [35].

The present study is not without limitations. The results of the present study only focus on effects of a recreational football intervention on functional movement performance. There are other potential outcomes from engagement in an exercise intervention that may be useful to understand in future studies. For example, whether participation in recreational football enhances other aspects of movement performance such as gait speed, postural stability and motor skill, amongst others, would be a useful future research focus. The sample size within the present study might also be considered small. Recruiting for such a study is difficult, particularly when the concept of running football, as is the case in the current study, is not viewed as the norm, and may be considered challenging for potential participants by those partaking in it. The current study was powered to 70%, where a posteriori power analysis indicated that for a medium effect size at 80% power with *p* = 0.05, a total sample size of 34 participants was needed. As a result, the current study was slightly underpowered. Similarly, whether the intervention had any effect on cardiovascular health or indices such as resting blood pressure, heart rate or heart rate variability would be worthy of examination in future research. There is also the possibility that participation in recreational football, due to its group-based nature, might influence psychosocial and mental health outcomes in an older adult population. This possibility was not examined in the present study but would be an interesting future avenue for research. Likewise, we assessed the intensity of the recreational football sessions that participants undertook using RPE. This indicated that the recreational football sessions were largely considered as ‘moderate’ in nature. There was variation within participants and depending on the stage of the intervention duration, with RPE scores ranging from 10 (light) to 15 (hard) within and across sessions. The concept of recreational football relates to self-selection of intensity based on the individual’s physical ability [1], so it is logical to assume we may see differences in intensity as the nature of involvement for participants should be to do what they are capable of doing and not push themselves further (to avoid injury by overreaching their capacity). While RPE is a valid measure of individual perception of exercise intensity, using other metrics, such as heart rate or global-positioning-system-related metrics, would be a useful next step. Such an approach would provide some further detail to understanding if all participants engaged with the same or similar physical effort in the sessions as well as objectively characterising the load involved in engaging in recreational football. A direct comparison between walking and recreational football in exploring the ‘load’ older adults experience when participating in recreational football would also be useful for researchers and practitioners to understand the benefits of recreational over walking football for health.

## 5. Conclusions

This study demonstrates that recreational football, as a health-enhancing intervention in older adults, results in significant improvement in functional movement reflective of lower body muscular endurance, agility and aerobic endurance performance.

## Figures and Tables

**Table 1:** Mean (M), standard deviation (SD) and 95% confidence intervals (CIs) for anthropometric and functional fitness variables before and after in recreational football for health intervention group and matched controls.

	Intervention						Control							
	Pre			Post			Pre			Post			Pre–Post X Group Interactions	
	M	SD	95% CI	M	SD	95% CI	M	SD	95% CI	M	SD	95% CI	*p*	Pƞ^2^
Physical activity (MET.min.week)	3020.3	1178.0					3118.3	1310.2						
BMI (kg/m^2^)	28.8	5.4	26.5–31.2	28.6	5.4	26.2–30.9	26.4	2.6	23.1–27.8	26.4	2.4	23.0–27.8	>0.05	0.007
Body fat (%)	30.8	6.4	27.8–33.8	27.8	6.4	24.9–30.7	26.6	3.8	23.6–29.6	26.9	3.1	24.1–29.8	0.07	0.127
30 s chair stand (reps)	17.8	4.7	15.6–19.8	19.8	3.3	18.3–21.2	14.0	2.2	10.9–15.2	12.7	2.9	11.2–14.2	0.038	0.168
30 s arm curl test (reps)	23.5	3.9	21.6–25.3	23.9	4.2	21.8–25.8	14.2	2.2	12.4–16.1	15.3	2.8	13.3–17.3	>0.05	0.009
8-foot get up and go (secs)	5.4	0.5	5.0–5.7	4.1	0.5	3.7–4.6	5.5	0.6	5.2–5.8	5.6	0.7	5.2–5.9	0.001	0.577
6 min walk test (m)	557.9	71.3	519.9–595.8	623.1	82.8	570.4–675.8	557.2	61.0	518.2–590.1	530.5	100.6	477.7–583.2	0.036	0.171
Grip strength (kg)	36.6	9.1	31.7–41.6	37.5	6.9	32.9–42.1	32.5	7.9	27.6–37.4	31.3	9.0	26.7–35.8	>0.05	0.011

## Data Availability

Not applicable.
